# Small residual patent ductus arteriosus after surgical ligation in young adult: to close or not to close - a dilemmatic case report

**DOI:** 10.11604/pamj.2021.38.219.22448

**Published:** 2021-02-25

**Authors:** Alexander Edo Tondas, Rido Mulawarman, Monica Trifitriana, Moza Guyanto, Raymond Pranata, Komaria

**Affiliations:** 1 Department of Cardiology and Vascular Medicine, Mohammad Hoesin General Hospital, Palembang, Sumatera Selatan, Indonesia,; 2 Biomedicine Doctoral Program, Faculty of Medicine, Universitas Sriwijaya, Palembang, Indonesia,; 3 Faculty of Medicine, Universitas Sriwijaya Palembang, Indonesia,; 4 Faculty of Medicine, Universitas Pelita Harapan, Tangerang, Indonesia

**Keywords:** Ligation, occluder, patent ductus arteriosus, residual, case report

## Abstract

Percutaneous transcatheter closure has gained acceptance for patent ductus arteriosus (PDA) management ever since its introduction, including the management residual left-to-right shunts following surgical ligations. It is preferred than the more invasive surgical closure. While large PDA is closed to prevent heart failure, the decision to close a small hemodynamically insignificant PDA is still a debatable issue. We present a case of percutaneous transcatheter closure of small residual left-to-right shunt PDA using HeartR™ Lifetech PDA occluder with instantaneous closure in an asymptomatic adult patient. The justification of closure was made based on the previous history of infective endocarditis, followed by PDA ligation and endarterectomy surgery, at 1.5 year before admission.

## Introduction

Residual shunt after patent ductus arteriosus (PDA) ligation has been reported to be roughly 6%, as a result from sub-optimal occlusion or recanalization of a totally occluded ductus [1]. Historically, infective endocarditis (IE) was a rare but often fatal complication of PDA, therefore, closure of hemodynamically insignificant small PDA is still a debatable issue [2,3]. Despite the successes of the first approved device Amplatzer™ duct occluder (ADO), new devices with different structural characteristics have been produced [4,5]. In this case, the HeartR™ PDA occluder (Lifetech Scientific Co, Ltd., Shenzhen, China) was used to close a small residual PDA in a young adult.

## Patient and observation

A 28-year-old male patient with a history of premature birth had undergone PDA ligation and endarterectomy 1.5 years ago on a large, type A, left-to-right shunt PDA with 7 mm diameter, which was diagnosed later in adulthood, due to lack of awareness. The decision for open heart surgery was made in consideration of vegetation growth in the main pulmonary artery (MPA) side of the PDA noticed by echocardiography, and obvious clinical signs of definite infective endocarditis. After the procedure, left ventricular (LV) ejection fraction was 59% and functional class improved from New York Heart Association (NYHA) III to NYHA I. Medications to control heart failure such as angiotensin converting enzyme (ACE) inhibitor, beta blocker and spironolactone was titrated according to clinical development.

**Figure 1:**
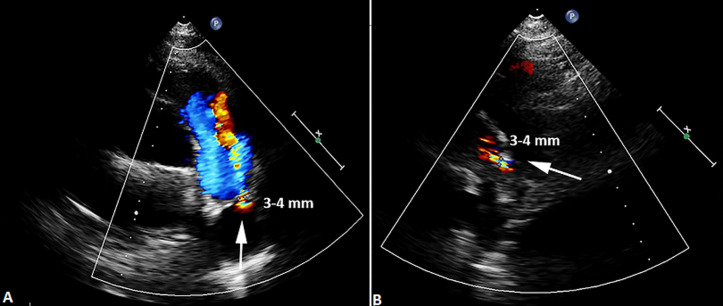
two-dimensional echocardiography showed the small residual PDA

**Figure 2:**
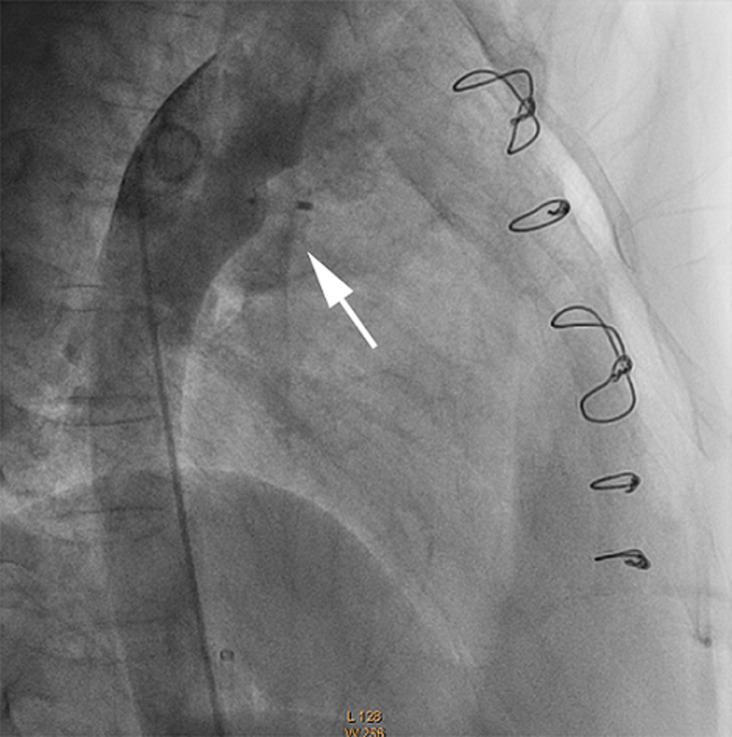
final angiography post PDA closure with HeartR™ device

The patient came back to us for his annual check-up visit, and was presented without any notable symptom except atypical chest discomfort. However, 2-dimensional echo exam revealed a persistent small residual PDA, 3-4 mm in diameter, with left-to-right shunt (Figure 1). After informed consent, transcatheter device closure was planned in concern of IE relapse. Under sedation, antegrade access was obtained via the right femoral vein and retrograde access was obtained via the right femoral artery. An initial angiography was performed with a 5F pigtail catheter positioned in the proximal descending aorta with lateral and 30^o^ right anterior oblique (RAO) projections. A 0.035” wire was used to cross the PDA and delivered into the main pulmonary artery (MPA). Cardiac catheterization measurements calculated FR of 1, PARi of 1.13 WU/m^2^, and PVR/SVR of 0.32. Angiography from descending aorta (AoD) revealed type A PDA with 3.4 mm isthmus diameter and 17.31 mm ampulla size (Figure 2). Subsequently, 8/10 mm HeartR™ PDA occluder (Life-Tech Sciences) was deployed across PDA anterogradely. After confirming the shunt closure by two-dimensional echocardiogram and descending thoracic aortogram, the delivery system was removed retrogradely. The procedure was successful without any meaningful complication. Fluoroscopy time was 10 minutes and 29 seconds. Echocardiography evaluation directly after closure showed no residual shunt and acceptable device position. Post-procedural course was uneventful, and no residual PDA was observed until one year follow up of this patient.

## Discussion

Patent ductus arteriosus in adults is a rare case compared to PDA in infants and early childhood with only 0,05% prevalence [6]. Residual shunts in post ligation PDA can occur in approximately 6% of cases. Even though spontaneous closure of silent PDA might occur within 3-6 months, 7% of patients will require a second procedure to close the shunt [7]. Patients with a small and hemodynamically insignificant PDA often remain asymptomatic and may never develop symptoms. However, regardless of the size, complications may arise; most importantly IE. As previously mentioned, the incidence of IE in PDA is very rare; however, this incidence may be higher in some developing countries due to nonexistent mechanisms of prevention, delay in seeking treatment due to poor financial status, lack of a proper health delivery system, and poor knowledge of how to treat fever in patients with underlying heart disease [8]. The decision to close small and hemodynamically insignificant PDA for the sole purpose of preventing the very rare incidence of IE is still debatable with many authors. Many suggest not to close the small PDA due to the rare incidence of IE and higher risk than benefit, while others prefer to close the PDA since IE is a fatal complication that can result in patient´s death if not treated adequately and in time. Infective endocarditis is also responsible for almost half of deaths in patients with untreated PDA [3,9]. Considering the fatality of IE should it occur and the patient´s previous history of IE, we decided to close the PDA.

Closure of PDA in cardiac catheterization laboratory is preferred to surgery in most cases since the latter is more invasive and mandates the use of inhaled anesthetic agents for general anesthesia. Several factors determine the success of percutaneous device closure of PDA including vascular accessibility, size and morphology of the duct, and device selection [6]. Transcatheter closure of PDA usually employs either coils or nitinol-based devices [10]. Amplatzer Duct Occluder (ADO) is the most commonly used device with excellent occlusion rates (99%-100%) and a low incidence of complications (0-7%) [4]. In this case, we use HeartR™, the first generation of Lifetech PDA occluder as the device to close the small residual PDA. This device is made of 0.004 nitinol wire mesh and shaped similar to ADO; however, the polyester fabric in the ADO device was replaced by expanded polytetrafluoroethylene (ePTFE) membrane to promote instantaneous closure of the duct and thereby eliminate residual flows. The foaming of the blood through the polyester fabric of ADO usually results in a continued left-to-right color Doppler flow, which is difficult to differentiate from a residual flow, due to incomplete device apposition to the ampulla. The absence of blood foaming across ePTFE in HeartR^TM^ occluder aids the immediate echocardiographic assessment of device deployment. Even though the ePTFE material caused significant acoustic shadowing beyond the device giving poor views of the descending aorta; the identification of residual flows, if any, was easier in the anteriorly placed pulmonary arteries [10].

## Conclusion

In conclusion, the closure of a small residual PDA from previous surgical ligation may be justified in susceptible patients with past history of infective endocarditis. The usage of transcatheter devices made from ePTFE material can assist operators in the judgement of immediate closure after deployment.
